# Optimization Techniques to Deeply Mine the Transcriptomic Profile of the Sub-Genomes in Hybrid Fish Lineage

**DOI:** 10.3389/fgene.2019.00911

**Published:** 2019-10-30

**Authors:** Zhong Wan, Jiayi Tang, Li Ren, Yamei Xiao, Shaojun Liu

**Affiliations:** ^1^School of Mathematics and Statistics, Central South University, Changsha, China; ^2^State Key Laboratory of Developmental Biology of Freshwater Fish, Hunan Normal University, Changsha, China

**Keywords:** transcriptomic profile, distant hybridization, optimization model, algorithm, classification, hybrids of fish, nonnegative matrix factorization

## Abstract

It has been shown that reciprocal cross allodiploid lineage with sub-genomes derived from the cross of *Megalobrama amblycephala* (BSB) × *Culter alburnus* (TC) generates the variations in phenotypes and genotypes, but it is still a challenge to deeply mine biological information in the transcriptomic profile of this lineage owing to its genomic complexity and lack of efficient data mining methods. In this paper, we establish an optimization model by non-negative matrix factorization approach for deeply mining the transcriptomic profile of the sub-genomes in hybrid fish lineage. A new so-called spectral conjugate gradient algorithm is developed to solve a sequence of large-scale subproblems such that the original complicated model can be efficiently solved. It is shown that the proposed method can provide a satisfactory result of taxonomy for the hybrid fish lineage such that their genetic characteristics are revealed, even for the samples with larger detection errors. Particularly, highly expressed shared genes are found for each class of the fish. The hybrid progeny of TC and BSB displays significant hybrid characteristics. The third generation of TC-BSB hybrid progeny ($BT_{F_{3}}$ and $TB_{F_{3}}$) shows larger trait separation.

## Introduction

Taxonomy aims to define and name groups of biological organisms on the basis of their shared similarity in morphological structure and physiological functions (Tautz et al., 2002). It plays an important role in understanding the relationship and evolution between different groups (Tautz et al., 2003). From classical morphology to new achievements in modern molecular biology, taxonomy also involves the comprehensive application of biological multidisciplinary, which can be used as a basis for classification, such as chromosome-based cell taxonomy (or chromosomal taxonomy), serum taxonomy based on serum reaction, chemical composition-based chemical taxonomy, and DNA taxonomy, with the sequence analysis of a uniform target gene (Stoeckle, 2003).

In the past two decades, with an increasing number of genome-wide sequencing and fine mapping, extensive data on transcriptomics, proteomics and metabolomics are available in the literature (Liu et al., 2016; Ren et al., 2016; Ren et al., 2017a; Ren et al., 2017b; Floriou-Servou et al., 2018; Li et al., 2018; Wang L. et al., 2018; Wang M. et al., 2018; Wang N. et al., 2018; Ye et al., 2018; Chen et al., 2019; Liu et al., 2019; Ning et al., 2019). To mine more and more biological information from these data, many computational models have been established to classify different species or examine their genetic relationships (Yang et al., 2015;Tan et al., 2019). For example, in (Wang L. et al., 2018; Wang M. et al., 2018; Wang N. et al., 2018; Yu et al., 2015; Wang et al., 2017; Hu et al., 2012), some statistical methods and statistical softwares have been used for biological classification by analyzing the data of protein sequences. However, to our best knowledge, there exists no research result on classification of distant multi-generation hybrid fishes in virtue of transcriptome data and optimization techniques.

Distant hybridization is a hybrid between two different species (Lou and Li, 2006). For this interspecific hybridization, it may be a hybrid of different species of the same genus, or between different genus, between different subfamilies, between different families, and even between different individuals Zhang et al., 2014). Since distant hybridization can transfer a set of genomes from one species to another, it can effectively change the genotype and phenotype of hybrid progeny (Liu et al., 2001). In terms of genotype, distant hybridization can lead to changes in the genomic level and sub-genome levels of the offspring, and the formation of these different hybrid progeny often depends on the genetic relationship of the parent. In terms of phenotype, the distant hybridization can integrate the genetic characteristics of the parents, which may make hybrid progeny show heterosis in aspects of shape, growth rate, survival rate and disease resistance (Hu et al., 2012). It has been shown that the distant hybridization occurs widely in fishes and has become an effective tool to integrate existing natural species and quickly cultivate more excellent traits in fisheries development. For more details, readers are referred to recently published article (Qin et al., 2014; Hu et al., 2019) and the references therein.

Different from protein (DNA) sequences, the transcriptome of a cell or a tissue is the collection of RNAs transcribed in it, and is often dynamic and a good representative of the cellular state (Carnes et al., 2018). Ease of genome-wide profiling using sequencing technologies further makes the transcriptome analysis an important research tool of bioinformatics, where the information content of an organism is recorded in the DNA of its genome and expressed through transcription (Kaletsky et al., 2018). Therefore, full-length transcriptome analysis of distant multi-generation hybrid fishes seems to be a more useful tools to provide a more profound explanation for the biological performance of distant multi-generation hybrid fishes. However, on the one hand, cultivating new generation of hybrid fishes often needs more than one and a half years, hence collection of the relevant experimental data is difficult, such that only the small-size sample inference can be made (Rogoza, 2019). On the other hand, owing to a lack of effective classic statistical methods to analyze the small-size and full-length transcriptome sample data, genomic research on similarity of this species and its descendants based on optimization models is unavailable in the literature. Actually, since the full-length transcriptome data is associated with expressed levels of ten thousands genes, classification of small-size sample data becomes impossible by using existing statistical methods. In this paper, combining the RNA sequencing group data of distant hybrid progeny and parental types, we intend to develop a new method for the genetic regulation of the whole transcriptome to statistically analyze the distant hybrid progeny and its excellent germplasm selection.

Basically, our new research method originates from optimization techniques, called a nonnegative matrix factorization method (NMF). By this method, we attempt to approximately factorize the small-size and full-length transcriptome sample data of the distant multi-generation hybrid fishes such that their classification and the gene-expression characteristic of each class can be revealed. As a result, it is associated with solution of large-scale optimization problems with nonnegativity constraints. Therefore, we also aim to develop an efficient algorithm for solving this large-scale optimization problem.

Clearly, one of the challenges in this research lies in making statistical inference from the small-size samples. We have collected 24 samples (liver tissues) of the distant multi-generation hybrid fishes, which constitutes three different groups corresponding to the three sampling periods. Each group consist of 20093 genes expression levels of eight different fish. Actually, the classical statistical methods, such as *k*-mean clustering method and the principal component analysis (PCA), are inappropriate to analyze this type of data (8 samples with 20093 features). As stated in (El-Shagi, 2017; Ristic-Djurovic et al., 2018; Rogoza, 2019), if the size of samples is small, it is difficult to believe that the classical statistical methods cangive good prediction accuracy owing to bias of small-size samples. For the small-size samples, the existing main inference methods include: the probabilistic index models (Amorim et al., 2018), the bootstrapping U-statistics method (Jiang and Kalbfleisch, 2012), the Jackknife empirical likelihood inference (Zhao et al., 2015), the SVM-based methods (Cong et al., 2016), the grey-theory-based methods (Meng et al., 2017), and the neural network (Zhu et al., 2019). However, for the small-size samples with more than ten thousand features, such as the full-length transcriptome sample data of the distant multi-generation hybrid fishes, it is desirable to study new statistical inference methods to mine their statistical information.

The NMF has been regarded as a useful tool of unsupervised machine learning to classify the small-size samples with large-scale features (Pauca et al., 2006;Wan et al., 2018). It can integrate the functions of *k*-mean clustering method and PCA. However, the performance of NMF depends significantly on the development of efficient algorithms to solve the generated large-scale optimization problem such that the deviation of nonnegative matrix (sample data) factorization is minimized. Especially, if we need to classify 8 full-length transcriptome data of distant multi-generation hybrid fishes, it is necessary to factorize a matrix in *R* ^20093×8^. Suppose that there are *r* classes of fishes, then the number of design variables is 20093 × *r* + 8. For solving such a large-scale optimization model, it is still a challenge to develop an efficient algorithm. In this research, we intend to modify the spectral conjugate algorithm in (Deng et al., 2013) to solve the generated large-scale optimization problems. Our goal is to reveal the relationship between multi-generation hybrid fishes on the basis of their gene expression profile described by their transcriptome data.

## Materials and Methods

### Samples and Transcriptome Sequencing

The *Megalobrama amblycephala or *Bluntnose black bream (BSB, 2*n* = 48 ) and *Culture Alburnus or *Topmouth culter (TC, 2*n* = 48) at sexual maturity in natural waters of the Yangtse River in China were collected for creating the allodiploids BT (BSB (♀)× TC (♂) ) and TB ( TC (♀) × BSB (♂) ) F_1_ individuals through intergeneric reciprocal crosses of BSB and TC, respectively. Then, the allodiploid F_2_ − F_3_ (2*n* = 48) hybrid offspring were obtained by self-mating of F_1_ – F_2_ populations, respectively. The chimeric offspring was identified based on 45S rDNA sequencing characteristics (Xiao et al., 2016), had been used in our study.

### Transcriptome Sequencing and Gene Expression Profiles

To sequence the transcriptomes of reciprocal cross hybrids and their inbred parents, total RNA was isolated and purified from the liver by a TRIzol extraction method (Rio et al., 2010). RNA concentration was measured using Nanodrop technology. Total RNA samples were treated with DNase I (Invitrogen) to remove any contaminating genomic DNA. The purified RNA was quantified using a 2100 Bioanalyzer system (Agilent, Santa Clara, CA, USA). After the isolation of 1 μg mRNA using the beads with oligo (dT) Poly (A), fragmentation buffer was added for interrupting mRNA to short fragments. The resulting short fragments were reverse transcribed and amplified to produce cDNA. An Illumina RNA-seq library was prepared according to a standard high-throughput method ephigh-throughput method (Dillies et al., 2013). The cDNA library concentration and quality were assessed by the Agilent Bioanalyzer 2100 system, after which the library was sequenced with paired-end setting using the Illumina HiSeq 2000/4000 platform. Then, the raw reads containing adapters, ploy-N and low quality were removed using in-house perl scripts. The high quality reads were used in our analysis. The transcriptome data was obtained from the NCBI database.

All Illumina reads of *M. Amblycephala* and *C. alburnus* were aligned to the *M. Amblycephala* and *C. alburnus* genome using Star (v 2.4.0) with the default parameters (Bennett et al., 2001), respectively. The other RNA-seq reads of reciprocal cross hybrids were aligned to the two reference genomes of *M. Amblycephala* and *C. alburnus*, respectively. The numbers of mapping counts in each gene were calculated with in-house perl scripts. Consequently, the two mapping results of aligning to two reference genomes were obtained in hybrid offspring, and the total expression value was normalized based on ratio of the number of mapped reads at each gene to the total number of mapped reads for the entire genome.

### Data Download

The collected data of 24 samples (liver tissues) of the distant multi-generation hybrid fishes in this research have been uploaded to https://github.com/TJY0622/TJY and can be downloaded freely such that the numerical experiments in this paper can be repeated by anyone. The last upload time is 07-20-2019(File name as 2019_7_8 Copy.xlsx).

### An Optimization Model for Classifying the Hybrids Fishes

We first propose an optimization model for classifying the hybrids fishes on the basis of NMF. Mathematically, NMF is stated as follows. For a given matrix *A* ∈ *R^n^* ^×^ *^m^*, we need to decompose *A* into two nonnegative matrices *W* and *H*, i.e.

(2.1) A≈WH

where *W* ∈ *R^n^* ^×^ *^r^* and *H* ∈ *R^r^* ^×^ *^m^*. In particular, if the matrix *A* in (2.1) is the full-length transcriptome data of the distant multi-generation hybrid fishes, and *A* = *WH*, then *r* can represent the number of classes for this classification of fishes in the case that each column of *H* has only a unique element 1, while the other elements are zeros. Clearly, in this ideal case, the *k*-th column of *W* stands for the gene expression level of the *k*-th class of fishes, and its elements show the expression levels of different genes for each class. Therefore, *W* in Model (2.3) is called a base matrix in view of its practical meanings, while *H* is called a coordinate matrix.

For real sample data, it is often difficult to obtain the above ideal result of factorization. Therefore, we relax *A* = *WH* by *A* ≈ *WH*. In this case, each column of the matrix *A* is approximately equal to the linear combination of all column vectors of the matrix *W*, and the combination coefficients are given by the corresponding column vector of the matrix *H*, i.e. $A_{:,j}\approx \sum W_{:,k}\times h_{k,j}$, where *A*_:,_ *_j_* denotes the *j*-th column of the matrix *A*, *W*_:,_ *_k_* stands for the *k*-th column of the matrix *W*, and *h_k,j_* represents the element of the *k*-th row and the *j*-th column in the matrix *H*. In other words, $A=[A_{:,1},\ldots A_{:,m}]\in R^{n\times m}$, $W=[W_{:,1},\ldots W_{:,r}]\in R^{n\times r}$, and $H=[h_{k,j}]\in R^{r\times m}$.

Thus, if we define a membership matrix *R* ∈ *R^r^* ^×^ *^m^*:

(2.2) $$R_{i,j}=\frac{1}{n}\sum^{\text{​}}\frac{W_{:,i}\times h_{i,j}}{\sum_{k=1}^{r}W_{:,k}\times h_{k,j}},i=1,\ldots ,r;j=1,\ldots ,m.$$

Clearly, the *j*-th column of *R* represents the membership degrees of the *j*-th sample being affiliated all the different classes. Therefore, for all the samples, distinct differences of all the elements in each column of *R* imply an approximate classification result. By definition, the matrix *R* shows the result of classification in term of membership degrees, while each column of the matrix *H* exactly stands for the coordinate of each sample in the *r*-dimensional space linearly expanded by the *r* columns of *W*. In the case that all the *r* elements in each row of *W* have the same orders of magnitude, the classification results by *H* or *R* are same.

Unfortunately, it is very difficult to solve Problem (2.1) when *n* is very large, let alone the requirement of finding the unknown optimal number of classes *r*. To solve Problem (2.1), we first transform (2.1) into the following optimization model:

(2.3) $$\begin{array}{ll} \underset{W,H}{\text{min}} & F(W,H)=\frac{1}{2}{\left\|A-WH\right\|}_{F}^{2}\\ \text{s.t.} & W,H\geq 0, \end{array}$$

where ‖·‖*_F_* is the Frobenius norm. It has been shown that (2.3) is non-convex and NP-hard (Vavasis, 2009). Then, similar to the technique of alternating non-negative least squares (ANLS) in (Chu et al., 2004), we solve (2.3) by finding the optimal solutions of the following two convex sub-problems:

(2.4) $$W^{\left(k+1\right)}=\text{arg}\underset{W\geq 0}{\min}F\left(W,H^{\left(k\right)}\right),$$

(2.5) $$H^{\left(k+1\right)}=\text{arg}\underset{H\geq 0}{\min}F\left(W^{\left(k+1\right)},H\right).$$

It is noted that the above model of NMF was first proposed in (Paatero and Tapper, 1994). Summarily, there are two types of algorithms to solve Model (2.3) (Lin, 2007): the multiplicative update (MU) method (Cai et al., 2010; Shang et al., 2012; Huang et al., 2018; Deng et al., 2019) and the technique of alternating non-negative least squares (ANLS) (Chu et al., 2004). For the ANLS, a main focus is on development of efficient algorithms to solve the subproblems (2.4) and (2.5). For example, the projected gradient (PG) method (Lin, 2007), the projected Newton method (Gong and Zhang, 2012), and the projected quasi-Newton method (Zdunek and Cichocki, 2006) have been reported to be efficient for solving the large-scale optimization model (2.3), although no one method has overwhelming advantage compared with the others.

Recently, Deng et al. (2013)proposed an efficient algorithm to solve general large-scale unconstrained optimizations, and they demonstrated that the numerical performance of this algorithm outperforms the similar ones available in the literature. In this paper, we intend to extend it into solution of the subproblems (2.4) and (2.5), which are two large-scale optimization problems with nonnegativity constraints.

### Development of Algorithm

We are now in a position to present an efficient algorithm to solve the subproblems (2.4) and (2.5). Since both of them are large scale (the size of the problem is over 80000), we will extend the spectral conjugate gradient algorithm in (Deng et al., 2013) to solve the subproblems (2.4) and (2.5). Actually, in our previous research, this algorithm has been implemented to solve more than 700 large-scale benchmark test problems, and has been shown that its numerical performance outperforms the similar ones available in the literature.

In need of modifying the developed algorithm in (Deng et al., 2013) such that it can be used to solve Model (2.3), we first define the gradients of *F *in (2.4) and (2.5) with respect to the matrices *W* and *H*, respectively.

By direct calculation, it is easy to see that for any *i* and *j*,

(2.6) $$F_{W_{ij}}(W,H)=\frac{\partial F}{\partial W_{ij}}={\left(-AH^{T}+WHH^{T}\right)}_{ij},i=1,\ldots ,n;j=1,\ldots ,r$$

(2.7) $$F_{H_{ij}}(W,H)=\frac{\partial F}{\partial H_{ij}}={\left(-W^{T}A+W^{T}WH\right)}_{ij},i=1,\ldots ,n;j=1,\ldots ,r.$$

Then, we denote the following two matrices the gradients of *F*(*W, H*) with respect to the matrices *W* and *H*, respectively: 

(2.8) $$\nabla_{W}F(W,H)=-AH^{T}+WHH^{T},\;\nabla_{H}F(W,H)=-W^{T}A+W^{T}WH.$$

For two given matrices *S* and *T* with the same size, we define their inner product by

$$\langle S,T\rangle =\underset{i,j}{\sum }S_{i,j}\times T_{i,j}.$$

Then, for *k* = 0, a search direction of *F* at a given initial point *W* ^(0)^ is

(2.9) $$D_{0}=-\nabla_{W}F(W^{\left(0\right)},H^{\left(0\right)})=A{\left(H^{\left(0\right)}\right)}^{T}-W^{\left(0\right)}H^{\left(0\right)}{\left(H^{\left(0\right)}\right)}^{T}.$$

And for *k* ≥ 1, we define four matrices:

(2.10) $$\begin{array}{c} s_{k-1}=W^{\left(k\right)}-W^{\left(k-1\right)},\\ G_{W}^{\left(k\right)}=\nabla_{W}F(W^{\left(k\right)},H^{\left(k\right)}),\\ y_{k-1}=G_{W}^{\left(k\right)}-G_{W}^{\left(k-1\right)},\\ \bar{y_{k-1}}=y_{k-1}-G_{W}^{\left(k\right)}\frac{\langle G_{W}^{\left(k\right)},y_{k-1}\rangle }{{\left\|G_{W}^{\left(k\right)}\right\|}^{2}}, \end{array}$$

where *H* ^(^ *^k^* ^)^, *W* ^(^ *^k^* ^)^ and *W* ^(^ *^k^* ^− 1)^ are two given matrices. Similar to (Deng et al., 2013), we compute the spectral parameter and conjugate parameter by 

(2.11) $$\theta_{k}=\{\begin{array}{cc} \frac{\langle D_{k-1},y_{k-1}\rangle -\langle D_{k-1},G_{W}^{\left(k\right)}\rangle \frac{\langle G_{W}^{\left(k\right)},s_{k-1}\rangle }{{\left\|G_{W}^{\left(k\right)}\right\|}^{2}}}{\langle D_{k-1},\bar{y_{k-1}}\rangle }, & \text{if }\langle D_{k-1},\bar{y_{k-1}}\rangle >\eta {\left\|G_{W}^{\left(k-1\right)}\right\|}^{2},\\ \frac{\langle D_{k-1},y_{k-1}\rangle -\langle D_{k-1},G_{W}^{\left(k\right)}\rangle \frac{\langle G_{W}^{\left(k\right)},G_{W}^{\left(k-1\right)}\rangle }{{\left\|G_{W}^{\left(k\right)}\right\|}^{2}}}{\langle -D_{k-1},G_{W}^{\left(k-1\right)}\rangle }, & \text{otherwise,} \end{array}$$

And

(2.12) $$\beta_{k}=\{\begin{array}{cc} \frac{\langle G_{W}^{\left(k\right)},y_{k-1}\rangle -\langle G_{W}^{\left(k\right)},s_{k-1}\rangle }{\langle D_{k-1},\bar{y_{k-1}}\rangle }, & \text{if }\langle D_{k-1},\bar{y_{k-1}}\rangle >\eta {\left\|G_{W}^{\left(k-1\right)}\right\|}^{2},\\ \frac{\langle G_{W}^{\left(k\right)},y_{k-1}\rangle }{{\left\|G_{W}^{\left(k-1\right)}\right\|}^{2}}, & \text{otherwise,} \end{array}$$

where *D_k_*_−1_ is the search direction at *W* ^(^ *^k^* ^−1)^, determined by

(2.13) $$D_{k}=\{\begin{array}{cc} D_{0}, & \text{if }k=0,\\ -\theta_{k}G_{W}^{\left(k\right)}+\beta_{k}D_{k-1}, & \text{if }k>0. \end{array}$$

The following algorithm is developed to solve the subproblem (2.4) with the given *H* ^(^ *^k^* ^)^.

**Algorithm 1:** (Modified Spectral Conjugate Gradient Algorithm)

**Step 0 (Initialization).** Given constants 0 < *δ*_1_, *η*, *ρ* < 1, 0 < *δ*_2_, ε. Choose an initial matrix *W* ^(0)^ ∈ *R^n^* ^ × ^ *^r^*. *Set* *k*: = 0. **Step 1 (Search direction).** If $\left\|G_{W}^{\left(k\right)}\right\|\leq \epsilon$, then the algorithm stops. Otherwise, compute *D_k_* by (2.9) and (2.13). **Step 2 (Step length).** Determine a step length $\alpha_{k}=\max\{a_{l}|a_{l}=\rho^{l},l=0,\;1,\;2,\ldots ,\}$ such that α*_k_* satisfies the following inequality: $F(W^{\left(k\right)}+\alpha_{k}D_{k},H^{\left(k\right)})\leq F(W^{\left(k\right)},H^{\left(k\right)})+\delta_{1}\alpha_{k}\langle G_{W}^{\left(k\right)},D_{k}\rangle -\delta_{2}\alpha_{k}^{2}{\left\|D_{k}\right\|}^{2},$ (2.14) where ${\left\|D_{k}\right\|}^{2}=\overset{n}{\underset{i=1}{\sum }}\overset{r}{\underset{j=1}{\sum }}{\left(D_{k}\right)}_{ij}^{2}.$ **Step 3 (Update).** *Set* $W^{\left(k+1\right)}:\;=W^{\left(k\right)}+\alpha_{k}D_{k}$ and *k* : = *k* + 1. Return to Step 1.

Similarly, to solve the subproblem (2.5), we only need replace *W* and *H* by *H* and *W* in **Algorithm 1**, respectively. Particularly, we need to compute

$$\begin{array}{c} s_{k-1}=H^{\left(k\right)}-H^{\left(k-1\right)},\\ G_{H}^{\left(k\right)}=\nabla_{H}F(W^{\left(k\right)},H^{\left(k\right)}),\\ y_{k-1}=G_{H}^{\left(k\right)}-G_{H}^{\left(k-1\right)},\\ \bar{y_{k-1}}=y_{k-1}-G_{H}^{\left(k\right)}\frac{\langle G_{H}^{\left(k\right)},y_{k-1}\rangle }{{\left\|G_{H}^{\left(k\right)}\right\|}^{2}}, \end{array}$$

and

(2.15) $$D_{k}=\{\begin{array}{ll} D_{0}, & \text{if }k=0,\\ -\theta_{k}G_{H}^{\left(k\right)}+\beta_{k}D_{k-1}, & \text{if }k>0, \end{array}$$

where

$$\theta_{k}=\{\begin{array}{cc} \frac{\langle D_{k-1},y_{k-1}\rangle -\langle D_{k-1},G_{H}^{\left(k\right)}\rangle \frac{\langle G_{H}^{\left(k\right)},s_{k-1}\rangle }{{\left\|G_{H}^{\left(k\right)}\right\|}^{2}}}{\langle D_{k-1},\bar{y_{k-1}}\rangle }, & \text{if }\langle D_{k-1},\bar{y_{k-1}}\rangle >\eta {\left\|G_{H}^{\left(k-1\right)}\right\|}^{2}\text{​},\\ \frac{\langle D_{k-1},y_{k-1}\rangle -\langle D_{k-1},G_{H}^{\left(k\right)}\rangle \frac{\langle G_{H}^{\left(k\right)},G_{H}^{\left(k-1\right)}\rangle }{{\left\|G_{H}^{\left(k\right)}\right\|}^{2}}}{\langle -D_{k-1},G_{H}^{\left(k-1\right)}\rangle }, & \text{otherwise,} \end{array}$$

and

$$\beta_{k}=\{\begin{array}{cc} \frac{\langle G_{H}^{\left(k\right)},y_{k-1}\rangle -\langle G_{H}^{\left(k\right)},s_{k-1}\rangle }{\langle D_{k-1},\bar{y_{k-1}}\rangle } & \text{if }\langle D_{k-1},\bar{y_{k-1}}\rangle >\;\eta {\left\|G_{H}^{\left(k-1\right)}\right\|}^{2},\\ \frac{\langle G_{H}^{\left(k\right)},y_{k-1}\rangle }{{\left\|G_{H}^{\left(k-1\right)}\right\|}^{2}}, & \text{otherwise,} \end{array}$$

With the above preparation, we now develop an overall algorithm to solve Model (2.3) in the end of this section.

**Algorithm 2:** 

**Step 0 (Initialization)**. Randomly generate two initial non-negative matrices *W * ^(0)^ ∈ *R^n × r^* and *H* ^(0)^ ∈ *R^r × m^*. Take constants $\delta_{1}^{W}$, $\delta_{1}^{H}$, *η^W^*, *η^H^*, *ρ^W^*, *ρ^H^* in the interval (0,1). Choose $0<\delta_{2}^{W},\delta_{2}^{H},\in$. Then, set *k*: = 0.
**Step 1 (Judgement)**. If $KKT(\bar{W^{\left(k\right)}},\bar{H^{\left(k\right)}})\leq \epsilon KKT(W^{\left(0\right)},H^{\left(0\right)})$, where *KKT* denotes the KKT conditions of Problem (2.1), and *KKT*(*W*, *H*) denotes the value of *KKT* at the matrix *W* and *H*. Then, this algorithm stops.
**Step 2 (Solution of Subproblem (2.4))**. Solve the subproblem (2.4) with $H=\bar{H^{\left(k\right)}}$ by Algorithm 1, its optimal solution is referred to as *W* ^(^ *^k^* ^ + 1)^.
**Step 3 (Projection of ** ***w*** **)**. Replace *W* ^(^ *^k^* ^ + 1)^ by (2.16) $$\bar{W_{i,j}^{\left(k+1\right)}}=\{\begin{array}{ll} 0, & if\;W_{i,j}^{\text{(}k+1)}<\;0,\\ W_{i,j}^{\left(k+1\right)}, & \text{ otherwise }, \end{array}i=1,\ldots ,n;j=1,\ldots ,m.$$
**Step 4 (Solution of Subproblem (2.5))**. Solve the subproblem (2.5) with $W=\bar{W^{\left(k+1\right)}}$ by Algorithm 1. The optimal solution is referred to as *H* ^(^ *^k^* ^ + 1)^.
**Step 5 (Projection of *H*)**. Replace *H* ^(^ *^k^* ^ + 1)^ by (2.17) $$\bar{H_{i,j}^{\left(k+1\right)}}=\{\begin{array}{ll} 0, & \text{if }H_{i,j}^{\left(k+1\right)}<\;0,\\ H_{i,j}^{\left(k+1\right)}, & \text{ otherwise }, \end{array}i=1,\ldots ,n;j=1,\ldots ,m.$$ **Step 6 (Update)**. Set *k* := *k* + 1. Go to Step 1. **Remark 1** Compared with the similar algorithms available in the literature (Li and Wan, 2019), Algorithms 1 and 2 present a different computational procedure to solve Problem (2.1). Since the existing nonnegative matrix factorization methods depends on development of efficient solution algorithms, one of our contributions in this paper lies in developing Algorithms 1 and 2 to solve a sequence of subproblems like (2.4) and (2.5). Especially, in the section of result, we will implement them to solve the classification problem of distant multi-generation hybrid fishes based on their transcriptome profiles. **Remark 2 **In order to improve efficiency of Algorithm 2, before factorization of *A*, we conduct normalization of the sample data of fishes as follows. (2.18) $$b_{i}=\underset{1\leq k\leq m}{\max}A_{i,k},i=1,\ldots ,n.$$ (2.19) $$a_{i}=\underset{1\leq k\leq m}{\min}A_{i,k},i=1,\ldots ,n.$$ (2.20) $$AA_{i,:}=\frac{A_{i,:}-a_{i}}{b_{i}-a_{i}},i=1,\ldots ,n.$$ where *A* ∈ *R^n^* ^ × ^ *^m^,* *A_i,j_* denotes the element of the *i*-th row and the *j*-th column in the matrix *A*, *AA_i,:_* denotes all the elements of the *i*-th row of the matrix *A*. **Remark 3** In Algorithm 2, since it is possible that the sequences $\left\{\bar{W^{\left(k\right)}}\right\}$ and $\left\{\bar{H^{\left(k\right)}}\right\}$ are trapped near a curved valley, we take $KKT(\bar{W^{\left(k\right)}},\bar{H^{\left(k\right)}})\leq \epsilon KKT(W^{\left(0\right)},H^{\left(0\right)})$ as the termination condition, rather than $KKT(\bar{W^{\left(k\right)}},\bar{H^{\left(k\right)}})<\epsilon$.

## Results

In this section, in virtue of Model (2.3) and **Algorithm 2**, we present the results on classification of the distant multi-generation hybrid fishes based on their transcriptome data.

### Result Of Classification

With the given transcriptome data of the distant multi-generation hybrid fishes, we easily get Model (2.3). Then, we implement Algorithm 2 to solve this model by choosing the same values of algorithmic parameters as in (Deng et al., 2013):

$$\begin{array}{l} \varepsilon =10^{-7},\;\delta_{1}^{W}=\delta_{1}^{H}=0.4,\;\eta^{W}=\eta^{H}=0.001,\;\\ \delta_{2}^{W}=\delta_{2}^{H}=0.001,\;\rho^{W}=\rho^{H}=0.65. \end{array}$$

In addition, for any choice of, *ρ^W^*, *ρ^H ^*∊ [0.05, 0.75] we can obtain the almost same results in our numerical experiments, which indicates our algorithms are robust for classifying the fishes.

All codes of the computer procedures are written in MATLAB and run in a MATLAB R2016b, and are carried out on a PC(CPU 2.40 GHz,8G memory) with the Windows 10 operation system environment. All the codes have been uploaded to https://github.com/TJY0622/TJY.

For the sake of better understanding the inherent characteristics of the data, we take the 2nd-group samples with superscripts *L*_2_ as a training set, which were from the liver tissue of eight different fish. Since it is unclear how many classes can be identified for the fish samples before our research, we make a trial setting on the number of classes *r* = 2, …, 7 such that the best number of classes is found.

In **Table 1**, we report all the numerical results corresponding to the different class numbers.

**Table 1:** Coordinate matrices for the 2nd-group samples.

Number of distant multi-generation hybrid fishes								
Class	$BSB^{L_{2}}$	$BT_{F_{1}}^{L_{2}}$	$BT_{F_{2}}^{L_{2}}$	$BT_{F_{3}}^{L_{2}}$	$TB_{F_{1}}^{L_{2}}$	$TB_{F_{2}}^{L_{2}}$	$TB_{F_{3}}^{L_{2}}$	$TC^{L_{2}}$
*r* = 2								
1*st*	1525	2293	1646	2843	1302	1552	2060	0
2*nd*	0	0	4.290	0	7.198	6.461	10.57	40.79
*r* = 3								
1*st*	9304	3067	5655	0	2821	5609	1195	0
2*nd*	0	2181	743.4	3759	967.7	664.0	2353	0
3*rd*	0	0	1.435	0	2.146	1.957	2.932	11.28
*r* = 4								
1*st*	2342	0	355.2	107.4	343.5	919.9	89.23	0
2*nd*	6.080	494.8	0	2787	430.6	165.2	1474	0
3*rd*	0	0	0.2183	0	0.7572	0.7388	1.110	4.158
4*th*	0	4607	3888	20.74	1465	1704	1128	0
*r* = 5								
2*st*	0.0104	0.2348	0	1.070	0	0	0.1612	0.0024
2*nd*	210.5	0	36.08	0.3265	21.54	75.89	0	0
3*rd*	0	1412	1070	0	167.9	351.8	0	0
4*th*	0	0	181.0	0	500.3	290.1	841.0	0
5*th*	0	0	0.0571	0	0.0265	0.1295	0	1.425
*r* = 6								
1*st*	0	0	487.5	0	1267	0	1970	0
2*nd*	0.0196	0.4725	0	2.033	0	0	0.3370	0.0130
3*rd*	0	3249	2849	0	439.6	0	0	0
4*th*	0	4.336	0.3095	0	0	29.53	3.221	0
5*th*	0	0	0.0960	0	0.0493	0	0	1.876
6*th*	3.622	0	0.6887	0.0606	0.4646	0.0237	0	0
*r* = 7								
1*st*	0.0001	1.599	0.1299	0	0	0	0.0850	0.0030
2*nd*	0	0	0.0374	0	0.0076	1.769	0.0575	0.0002
3*rd*	0	0	0	0	0.0044	0	0	0.1940
4*th*	0	0	0	0	78.41	0	189.0	0
5*th*	4.695	0	0	0	0.2324	0	0	0
6*th*	0	0	272.2	0	103.1	0	0	0
7*th*	0	0	0	1.767	0	0	0.0475	0

**Table 1** shows that when *r* = 6, all the samples are clearly classified owing to existence of greater deviation of elements in the same column of *H*. In contrast, when *r* is equal to the other values, there are at least one sample that can not be clearly classified. 

As *r* = 6, **Table 1** indicates that the eight fishes can be categorized into 6 classes: $BSB^{L_{2}}$, $TC^{L_{2}}$, $TB_{F_{2}}^{L_{2}}$ and $BT_{F_{3}}^{L_{2}}$ belong to different four classes, respectively. $BT_{F_{1}}^{L_{2}}$ and $BT_{F_{2}}^{L_{2}}$ consist in another class. $TB_{F_{1}}^{L_{2}}$ and $TB_{F_{3}}^{L_{2}}$ are the same class.

**Figure 1:**
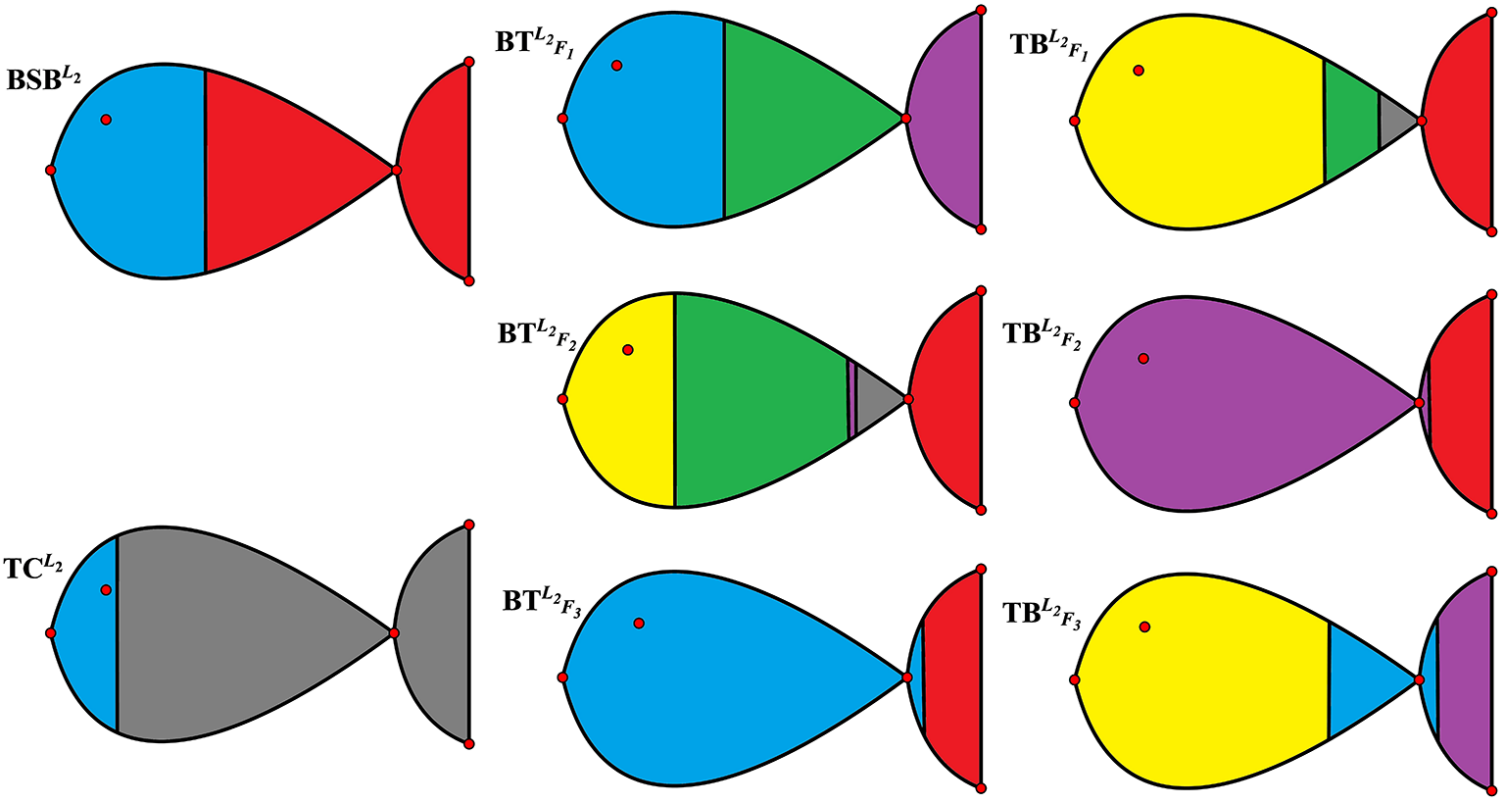
Membership degrees of the 2nd-group samples.

For the sake of better understanding the above classification result, we use *r* = 6 as the number of classes to calculate the membership matrix *R* defined by (2.2). The numerical results are listed in **Table 1**, while **Figure 1** more intuitively describe the biological similarity for the fish of each class.

**Table 2** and **Figure 1** further indicate that by membership matrices, the same classification result is obtained as that by coordinate matrices: $\left\{BSB^{L_{2}}\right\}$; $\left\{TC^{L_{2}}\right\}$; $\left\{TB_{F_{2}}^{L_{2}}\right\}$; $\left\{BT_{F_{3}}^{L_{2}}\right\}$; $\left\{BT_{F_{1}}^{L_{2}},BT_{F_{2}}^{L_{2}}\right\}$; $\left\{TB_{F_{1}}^{L_{2}},TB_{F_{3}}^{L_{2}}\right\}$. Particularly, either by *H* or by *R*, $BSB^{L_{2}}$ and $TC^{L_{2}}$ always belong to two different classes, while their hybrids are divided into different classes from the parents’ ones. In **Figure 1**, Classes from 1 to 6 are described by the colors of yellow, blue, green, purple, gray and red, respectively. It follows from **Figure 1** that larger proportion of the green color in $BT_{F_{1}}^{L_{2}}$ and $BT_{F_{2}}^{L_{2}}$ (that of the yellow color in $TB_{F_{1}}^{L_{2}}$ and $TB_{F_{3}}^{L_{2}}$) demonstrate that there exists greater degree of biological similarity between $BT_{F_{1}}^{L_{2}}$ and $BT_{F_{2}}^{L_{2}}$ (between $TB_{F_{1}}^{L_{2}}$ and $TB_{F_{3}}^{L_{2}}$).

**Table 2:** Membership matrix *R* of the 2^nd^-group samples.

Class	$BCB^{L_{2}}$	$BT_{F_{1}}^{L_{2}}$	$BT_{F_{2}}^{L_{2}}$	$BT_{F_{3}}^{L_{2}}$	$TB_{F_{1}}^{L_{2}}$	$TB_{F_{2}}^{L_{2}}$	$TB_{F_{3}}^{L_{2}}$	$TC^{L_{2}}$
1*st*	0	0	0.2826	0	0.6049	0	0.6132	0
2*nd*	0.3713	0.3880	0	0.8713	0	0	0.2588	0.1597
3*rd*	0	0.4268	0.4047	0	0.1333	0	0	0
4*th*	0	0.1853	0.0080	0	0	0.8493	0.1280	0
5*th*	0	0	0.1329	0	0.1029	0	0	0.8403
6*th*	0.6287	0	0.1718	0.1287	0.1589	0.1507	0	0

To further test robustness of the above trained results, given *r* = 6, we choose the 1st-group and the 3rd-group samples (with superscripts *L*_1_ and *L*_3_, respectively) as two test sets to see whether the results are the same or not.

**Figure 2:**
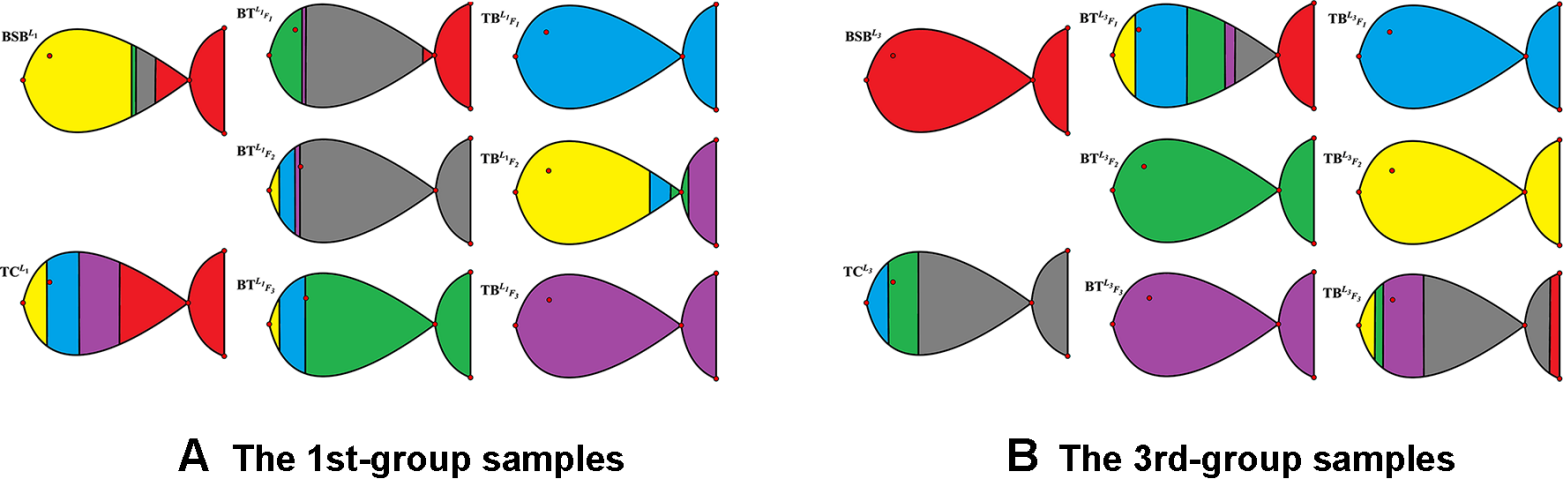
The membership degrees of 1st-/3rd-group samples.

In **Table 3** and **Figure 2**, we report the numerical results. The used colors in **Figure 2** only be used to show the similarity of fishes within the same figure. In other words, the same color has no any relation in different figures.

**Table 3:** Results for the 1st-/3rd-group samples.

Class	$BSB^{L_{1}}$	$BT_{F_{1}}^{L_{1}}$	$BT_{F_{2}}^{L_{1}}$	$BT_{F_{3}}^{L_{1}}$	$TB_{F_{1}}^{L_{1}}$	$TB_{F_{2}}^{L_{1}}$	$TB_{F_{3}}^{L_{1}}$	$TC^{L_{1}}$
Coordinate matrices of the 1st-group samples								
1*st*	521.9	0	24.01	2.318	0	1477	0	80.61
2*nd*	0	0	0.5099	0.1318	15.17	0.7512	0	0.9975
3*rd*	0.0016	0.5032	0	4.467	0	0.0197	0	0
4*th*	0	0.0751	0.0889	0	0	1.014	16.08	1.392
5*th*	0.0096	0.9609	2.580	0	0	0	0	0
6*th*	879.3	1865	0	0	0	0	0	3287
Membership matrices of the 1st-group samples								
1*st*	0.5442	0	0.0513	0.0468	0	0.6652	0	0.1244
2*nd*	0	0	0.0758	0.1370	1	0.1049	0	0.1573
3*rd*	0.0159	0.1592	0	0.8163	0	0.0946	0	0
4*th*	0	0.0122	0.0236	0	0	0.1353	1	0.2007
5*th*	0.1009	0.5906	0.8493	0	0	0	0	0
6*th*	0.3390	0.2380	0	0	0	0	0	0.5176
Class	$BSB^{L3}$	$BT_{F_{1}}^{L_{3}}$	$BT_{F_{2}}^{L_{3}}$	$BT_{F_{3}}^{L_{3}}$	$TB_{F_{1}}^{L_{3}}$	$TB_{F_{2}}^{L_{3}}$	$TB_{F_{3}}^{L_{3}}$	$TC^{L_{3}}$
Coordinate matrices of the 3rd-group samples								
1*st*	0	1.304	0	0	0	12.29	1.047	0
2*nd*	0	937.0	0	0	2013	0	0	180.7
3*rd*	0	0.7632	6.497	0	0	0	0.1616	0.6317
4*th*	0	0.0314	0	2.426	0	0	0.4113	0
5*th*	0	224.9	0	0	0	0	2609	2531
6*th*	1218	378.6	0	0	0	0	46.55	0
Membership matrices of the 3rd-group samples								
1*st*	0	0.1121	0	0	0	1	0.0778	0
2*nd*	0	0.2622	0	0	1	0	0	0.1117
*3rd*	0	0.1874	1	0	0	0	0.0384	0.1531
4*th*	0	0.0453	0	1	0	0	0.2062	0
5*th*	0	0.1418	0	0	0	0	0.6330	0.7352
6*th*	1	0.2511	0	0	0	0	0.0446	0

From **Table 3** and **Figure 2**, it is clear that 6 out of 8 samples in the 1st-group or the 3rd-group are correctly classified, compared with the trained result from the samples of the 2nd-group. The accuracy rate reaches 75%. In **Table A3**, we show that the elements in each row of the matrix *W* have different orders of magnitude for the 1st-group samples, which can explain inconsistence of the classification results by *H* and *R* for the 4 samples: ${\text{BSB}}^{L_{1}},{\text{BT}}_{F_{1}}^{L_{1}},{\text{BT}}_{F_{2}}^{L_{1}}$ and $BT_{F_{3}}^{L_{1}}$.

To further validate the proposed model and algorithms in this paper, we use them to classify more test samples generated by mixing the training set and the test sets.

We first mix the training set and the 3rd-group test set. The obtained results are listed in **Table 4**. **Table 4** demonstrates that compared with the trained result, 13 out of 16 samples are correctly classified by both of the membership and coordinate matrices, which includes all the samples in the 2nd-group and the 5 samples in the 3rd-group: ${\text{BSB}}^{L_{3}},{\text{BT}}_{F_{2}}^{L_{3}},{\text{BT}}_{F_{3}}^{L_{3}},TB_{F_{2}}^{L_{3}}$ and $TB_{F_{3}}^{L_{3}}$. The accuracy rate is as high as 81.25%. Additionally, for the 5 species of fish (BSB, ${\text{BT}}_{F_{\text{2}}}$, ${\text{BT}}_{F_{3}}$, ${\text{TB}}_{F_{\text{2}}}$ and ${\text{TB}}_{F_{3}}$), the replicated samples of each fish are correctly classified into the same class in our test experiments, which also validates the proposed model and algorithms in this paper.

**Table 4:** Results for the mixed samples of the 2nd/3rd group.

Coordinate matrices								
Class	$BSB^{L_{2}}$	$BSB^{L_{3}}$	$BT_{F_{1}}^{L_{2}}$	$BT_{F_{1}}^{L_{3}}$	$BT_{F_{2}}^{L_{2}}$	$BT_{F_{2}}^{L_{3}}$	$BT_{F_{3}}^{L_{2}}$	$BT_{F_{3}}^{L_{3}}$
1*st*	0	0.119	0.0451	0.0984	0	0	0.0208	0.0486
2*nd*	0	169.6	2662	17.07	1801	2057	0	27.20
3*rd*	0.4607	0	0.5200	0.0245	0	0.3763	2.565	2.5614
4*th*	0	139.7	0	545.9	129.5	3.907	0	3.533
5*th*	2719	2378	0	922.6	669.6	276.0	0	0
6*th*	0	0.4276	0	1.414	1.342	1.940	0.1942	0.0491
Class	$TB_{F_{1}}^{L_{2}}$	$TB_{F_{1}}^{L_{3}}$	$TB_{F_{2}}^{L_{2}}$	$TB_{F_{2}}^{L_{3}}$	$TB_{F_{3}}^{L_{2}}$	$TB_{F_{3}}^{L_{3}}$	$TC^{L_{2}}$	$TC^{L_{3}}$
1*st*	0	0	0.4164	0.4112	0	0	3.993	0.1466
2*nd*	80.51	0.7667	803.5	745.8	0	0	0	1016
3*rd*	0.0997	0	0.0059	0	0.0094	0	0	0
4*th*	206.2	938.5	0	55.71	0	7.602	3.805	106.1
5*th*	633.8	0	1572	1635	215.5	184.9	0	0
6*th*	3.514	0	1.430	0.6670	7.588	8.066	0	4.545
Membership matrices								
Class	$BSB^{L_{2}}$	$BSB^{L_{3}}$	$BT_{F_{1}}^{L_{2}}$	$BT_{F_{1}}^{L_{3}}$	$BT_{F_{2}}^{L_{2}}$	$BT_{F_{2}}^{L_{3}}$	$BT_{F_{3}}^{L_{2}}$	$BT_{F_{3}}^{L_{3}}$
1*st*	0	0.1093	0.0705	0.0813	0	0	0.0702	0.0894
2*nd*	0	0.1001	0.6086	0.0140	0.4571	0.4481	0	0.0340
3*rd*	0.4360	0	0.3210	0.0256	0	0.1797	0.8340	0.8241
4*th*	0	0.1616	0	0.3274	0.1147	0.0102	0	0.0204
5*th*	0.5640	0.4885	0	0.2465	0.1746	0.0803	0	0
6*th*	0	0.1404	0	0.3052	0.2536	0.2818	0.0957	0.0322
Class	$TB_{F_{1}}^{L_{2}}$	$TB_{F_{1}}^{L_{3}}$	$TB_{F_{2}}^{L_{2}}$	$TB_{F_{2}}^{L_{3}}$	$TB_{F_{2}}^{L_{3}}$	$TB_{F_{3}}^{L_{3}}$	$TC^{L_{2}}$	$TC^{L_{3}}$
1*st*	0	0	0.1882	0.2061	0	0	0.9315	0.0855
2*nd*	0.0420	0.1985	0.2412	0.2507	0	0	0	0.2608
3*rd*	0.0720	0	0.0138	0	0.0406	0	0	0
4*th*	0.1629	0.8015	0	0.0580	0	0.0327	0.0685	0.0906
5*th*	0.1735	0	0.3064	0.3376	0.1043	0.0941	0	0
6*th*	0.5496	0	0.2504	0.1476	0.8550	0.8733	0	0.5632

Next, we compute the classification result of all 24 samples (8 samples in the training set, 16 samples in the two test sets). The results are given in **Table 5**. From **Table 5**, we know that 17 out of 24 samples are correctly classified by the membership matrix or the coordinate matrix, which excludes $BSB^{L_{1}},\;BT_{F_{1}}^{L_{1}},\;BT_{F_{2}}^{L_{1}},\;BT_{F_{1}}^{L_{3}},\;TB_{F_{1}}^{L_{1}},\;TB_{F_{1}}^{L_{3}}$ and $TC^{L_{3}^{}}$. The accuracy rate achieves 70.83%, compared with the trained results. In this test, for the 4 species of fish (BSB, $BT_{F_{3}},\;TB_{F_{2}}$ and $TB_{F_{3}}$), the replicated samples of each fish are correctly classified into the same class.

**Table 5:** Results for the mixed samples of all three groups.

Coordinate matrices								
Class	$BSB^{L_{1}}$	$BSB^{L_{2}}$	$BSB^{L_{3}}$	$BT_{F_{1}}^{L_{1}}$	$BT_{F_{1}}^{L_{2}}$	$BT_{F_{1}}^{L_{3}}$	$BT_{F_{2}}^{L_{1}}$	$BT_{F_{2}}^{L_{2}}$
1st	0	671.3	2229	11.60	1301	5250	0	2311
2nd	0.4599	1.978	0	1.329	1.977	0.0994	0	0
3rd	3648	9042	7691	1933	832.1	2782	0	2691
4th	690.1	0	593.0	0	7580	949.7	1376	5044
5th	0.7721	0	0.3744	8.097	0	0	18.35	0
6th	172.2	0	0	152.1	0	188.9	0	126.2
Class	$BT_{F_{2}}^{L_{3}}$	$BT_{F_{3}}^{L_{1}}$	$BT_{F_{3}}^{L_{2}}$	$BT_{F_{3}}^{L_{3}}$	$TB_{F_{1}}^{L_{1}}$	$TB_{F_{1}}^{L_{2}}$	$TB_{F_{1}}^{L_{3}}$	$TB_{F_{2}}^{L_{1}}$
1st	1110	0	123.9	221.6	0	2112	7793	0
2nd	1.421	10.46	10.86	10.92	1.504	0.3468	0.0952	0.6075
3rd	1541	70.23	0	0	0	1854	0	6045
4th	6029	256.2	292.7	313.2	7931	1914	870.3	1571
5th	0	0	0	0	0.1716	0	0.0828	0
6th	221.6	24.25	19.02	0	112.6	420.3	0	365.6
Class	$TB_{F_{2}}^{L_{2}}$	$TB_{F_{2}}^{L_{3}}$	$TB_{F_{3}}^{L_{1}}$	$TB_{F_{3}}^{L_{2}}$	$TB_{F_{3}}^{L_{3}}$	$TC^{L_{1}}$	$TC^{L_{2}}$	$TC^{L_{3}}$
1st	326.0	1437	72.05	0	11.29	232.5	3530	1687
2nd	0	0	0	0	0	0.1171	0.4372	0
3rd	6203	6205	0	0	0	3044	1001	0
4th	3084	2639	1890	1785	1988	415.9	0	4294
5th	0	0	0	0	0	1.252	2.323	0.1871
6th	233.9	128.4	908.8	1051	1080	402.1	656.6	523.1
Membership matrices								
Class	$BSB^{L_{1}}$	$BSB^{L_{2}}$	$BSB^{L_{3}}$	$BT_{F_{1}}^{L_{1}}$	$BT_{F_{1}}^{L_{2}}$	$BT_{F_{1}}^{L_{3}}$	$BT_{F_{2}}^{L_{1}}$	$BT_{F_{2}}^{L_{2}}$
1st	0	0.1364	0.2535	0.0060	0.1487	0.3563	0	0.2087
2nd	0.0895	0.3355	0	0.1431	0.2199	0.0209	0	0
3rd	0.3139	0.5281	0.4873	0.1323	0.0767	0.2086	0	0.1864
4th	0.1110	0	0.1117	0	0.5546	0.1782	0.1522	0.4512
5th	0.2104	0	0.1475	0.5728	0	0	0.8478	0
6th	0.2752	0	0	0.1459	0	0.2360	0	0.1538
Class	$BT_{F_{2}}^{L_{3}}$	$BT_{F_{3}}^{L_{1}}$	$BT_{F_{3}}^{L_{2}}$	$BT_{F_{3}}^{L_{3}}$	$TB_{F_{1}}^{L_{1}}$	$TB_{F_{1}}^{L_{2}}$	$TB_{F_{1}}^{L_{3}}$	$TB_{F_{2}}^{L_{1}}$
1st	0.1046	0	0.0459	0.0722	0	0.1783	0.5848	0
2nd	0.1417	0.7920	0.7840	0.7989	0.1929	0.0497	0.0571	0.0823
3rd	0.1026	0.0226	0	0	0	0.1327	0	0.3242
4th	0.4424	0.1169	0.1187	0.1289	0.5787	0.2390	0.2783	0.2185
5th	0	0	0	0	0.0627	0	0.0799	0
6th	0.2087	0.0685	0.0514	0	0.1657	0.4003	0	0.3750
Class	$TB_{F_{2}}^{L_{2}}$	$TB_{F_{2}}^{L_{3}}$	$TB_{F_{3}}^{L_{1}}$	$TB_{F_{3}}^{L_{2}}$	$TB_{F_{3}}^{L_{3}}$	$TC^{L_{1}}$	$TC^{L_{2}}$	$TC^{L_{3}}$
1st	0.0518	0.1602	0.0275	0	0.0179	0.0325	0.1950	0.1457
2nd	0	0	0	0	0	0.0227	0.0487	0
3rd	0.3371	0.3534	0	0	0	0.2219	0.0634	0
4th	0.3345	0.3177	0.2540	0.2431	0.2457	0.0555	0	0.3488
5th	0	0	0	0	0	0.2311	0.2466	0.0586
6th	0.2765	0.1687	0.7185	0.7569	0.7364	0.4363	0.4464	0.4468

In summary, by all of the above test experiments, the average accuracy rate is 75.52% even if there exists larger detection error of the input initial sample data (see our subsequent correlation analysis). These tests further verifies that the proposed model and algorithm in this paper can be used to efficiently classify the distant multi-generation hybrid fishes based on their transcriptomic profile.

### Correlation Analysis

**Figure 3:**
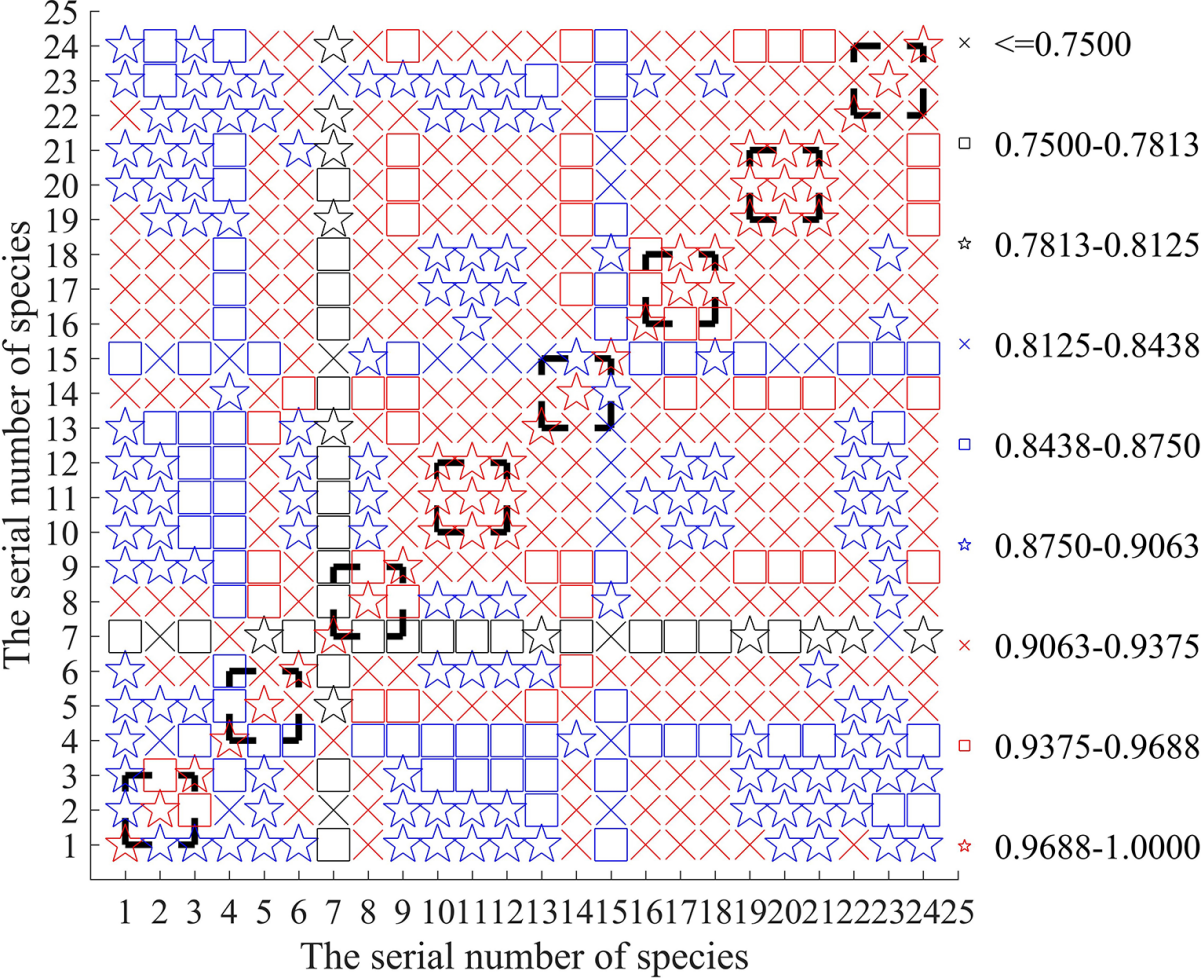
Correlation of the input 24 sample data.

To find out the reasons why the replicated samples are incorrectly classified such that the accuracy rate may be reduced, we calculate the correlation matrix of the sample data to reveal possible detection errors of the input initial data. In **Figure 3**, the correlation coefficient matrix of the 24 samples is concisely plotted.

From **Figure 3**, it is easy to see that the sample of $BSB^{L_{1}}$ is only weakly correlated with the two replicated samples $BSB^{L_{2}}$ and $BSB^{L_{3}}$. Their correlation degree is even less than that between the samples of different fish $BSB^{L_{1}}$ and $TB_{F_{2}}$. It can explain why $BSB^{L_{1}}$ can not be clearly classified into the same class of $BSB^{L_{2}}$ and $BSB^{L_{3}}$ (revisiting the results in **Table 5**). Conversely, **Figure 3** shows that in the 1st-group, the sample $BSB^{L_{1}}$ has greater correlation with the other 3 samples: $TB_{F_{2}}^{L_{1}},\;TB_{F_{3}}^{L_{1}}$ and $TC^{L_{1}}$, which answers why the class of $BSB^{L_{1}}$ can not be clearly identified in **Table 3**.

From **Figure 3**, we can also find out similar reasons for the unsatisfactory classification of $BT_{F_{1}}^{L_{1}},\;BT_{F_{1}}^{L_{2}},\;BT_{F_{1}}^{L_{3}}$ in **Tables 4** and **5**. Actually, (1) owing to lower correlation among $BT_{F_{1}}^{L_{1}},\;BT_{F_{1}}^{L_{2}}$ and $BT_{F_{1}}^{L_{3}}$, they can not be classified into the same class even if they are the three replicated samples. (2) In the 3rd group, the class of $BT_{F_{1}}^{L_{3}}$ can not be clearly identified in **Table 3** since its sample is more correlated with the other 5 samples: $BSB^{L_{3}},\;BT_{F_{2}}^{L_{3}},TB_{F_{1}}^{L_{3}},TB_{F_{2}}^{L_{3}}$ and $TC^{L_{3}}$.

Similarly, because the sample of $BT_{F_{2}}^{L_{1}}$ is only little correlated with the two replicated samples $BT_{F_{2}}^{L_{2}}$ and $BT_{F_{2}}^{L_{3}},\;BT_{F_{2}}^{L_{1}}$ can not be classified into the same class of $BT_{F_{2}}^{L_{2}}$ and $BT_{F_{2}}^{L_{3}}$ in **Table 5**.

For the same reason of weaker correlation, in **Tables 4** and **5**, the three replicated samples of $TB_{F_{1}}\;(TC)$ are also classified into the different classes. It is believed that if the detection errors of samples can be controlled to be small enough, the proposed model and algorithms in this paper can provide a more satisfactory result of classification. Actually, for the three species of fish: $TB_{F_{2}},\;TB_{F_{3}}$ and $BT_{F_{3}}$, their three replicated samples can always classified into the respective same class (see **Tables 4** and **5**), which may be related with higher correlation between them as shown in **Figure 3**.

### Genes Of High Expression

In the end of this section, based on our classification result from the 2nd-group samples, we answer what are the differently expressed genes in all the six classes. By definition, we know that each column of the base matrix *W* gives the feature of gene expression for each class of fish. Since the sample of each class consists of 20093 genes, we only list a part of the highly expressed genes for each fish. When *r* = 6, the highly expressed genes are reported in **Table A1** and **Figures 1(a), 1(b) and 1(c)**.

From the numerical results in **Table A1** and **Figures 1(a), 1(b) and 1(c)**, it follows that there exists stronger genetic similarity between the BSB (parents) and the hybrids. Actually, the BSB (the 6th class) has 3 shared highly expressed genes with $TB_{}$ (the 1st class), 45 shared highly expressed genes with $BT_{F_{3}}$ (the 2nd class) and 12 shared highly expressed genes with $TB_{}$ (the 4th class). In contrast, the TC (the 5th class) does not have any shared highly expressed genes with their hybrids, which implies that their hybrids seem to look more like BSB, rather than TC, regardless of reciprocal hybrids.

Apart from one-by-one comparison in **Table A1**, we also statistically analyze the numbers of shared highly expressed genes for more than three classes of fish. The reported results in **Table A2** demonstrate that BSB (6-th class) has higher hereditary conservatism than TC (5th class). Actually, by comparing the numbers of shared highly expressed genes among BSB, TC and the hybrids, it is clear that the gene expression profile of their grandchildren looks more like BSB (6st class), rather than TC (5th class).

It is also noted that in **Table A2**, there are no shared expressed genes between $BT_{F_{1}}$ (3rd class) and $TB_{F_{1}}$ (1st class), or between $BT_{F_{2}}$ (3rd class) and $TB_{F_{2}}$ (4th class), and there only exist 3 shared highly expressed genes between the $BT_{F_{3}}$ (2nd class) and $TB_{F_{3}}$ (1st class). It suggests that the trait separation occurs between these hybrids.

**Figure A1:**
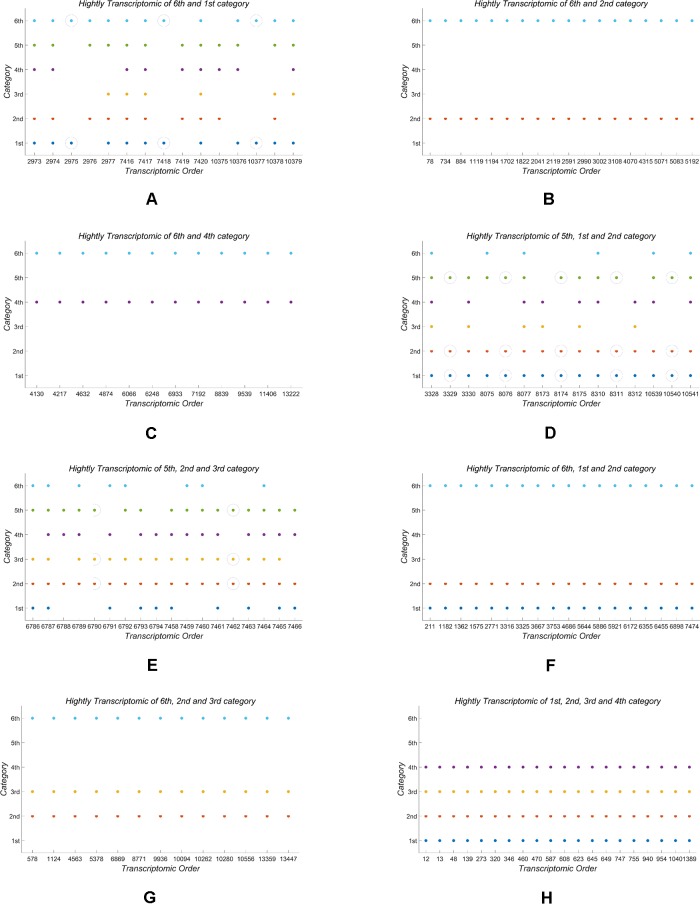
Shared highly expressed genes for the second-group samples.

In addition, from **Table A2** and **Figure A1**, it follows that the hybrids have larger transcript intersection than that between the hybrids and the parents, since the number of shared highly expressed genes between the hybrids (offspring) is far more than that between them and their parents. Actually, there are 277 shared highly expressed genes among $TB_{F_{3}},\;TB_{F_{1}}$ (1st class), $BT_{F_{3}}$ (2nd class) and $TB_{F_{2}}$ (4th class). In contrast, there are only less than 45 shared highly expressed genes between the parent (BSB) and the hybrids ($BT_{F_{3}}$).

## Discussion

In our numerical experiments, it is found that the nonnegative factorization of the matrix *A* is not unique. In particular, if we choose different initial matrices *W* ^0^ and *H* ^0^, the base and coordinate matrices *W* and *H* may be different. However, our numerical experiments show that for Algorithms 1 and 2, different choices of *W* ^0^ and *H* ^0^ do not affect the final result of classification. For example, as *r* = 6, the result of classification always is the same for any *W* ^0^ and *H* ^0^, which can show robustness of our classification method.

Hybridization is considered as the rapidly driving forces that shape epigenetic modifications in plants and parts of lower vertebrate (Liu et al., 2016; Mallet, 2005). The merge of divergent genome always results in a ‘genomic and transcriptome shock’ in newborn hybrid (Ren et al., 2017b; Wu et al., 2016; Ren et al., 2016). Analysis on the expression changes after hybridization, including expression dominance and expression bias related to specific function-regulated genes, always provides us insights into the molecule mechanism of various phenotypes including heterosis (Ren et al., 2016; Zhou et al., 2015). However, the multiple regulatory mechanism and complex protein interaction network restricted our ability to investigate the underlying regulation in hybrid.

It is noted that in this research, we choose the 2nd-group samples as the training set, instead of the 1st-group or 3rd-group, and the latter is regarded as test samples to verify the trained result. One of the reasons for our doing so lies in that correlation analysis of the three-group samples indicates that each sample in the second-group is better correlated with the other replicated samples than those in the other two groups.

The proposed model and algorithms in this paper can be extended to solve more practical engineering problems from other fields. For example, if we can collect sufficient transcriptome data of patients possibly suffering from breast cancer, we can apply the proposed model and algorithms to identify the classes of patients, even development of the relevant smart aided-system of diagnosis for the sufferers.

## Conclusions

In this paper, we have constructed a classification model for the distant multi-generation hybrid fishes based on transcriptome data, and developed an efficient algorithm, called the modified spectral conjugate gradient algorithm, for solving such a model.

In virtue of our model and algorithm, we have obtained a satisfactory classification for a given full-length transcriptome data of fish samples, and the differently expressed genes of each class have been identified. Our results are first obtained by a training set of samples, then are tested by many test samples generated by different ways.

Main results are stated as follows.

1. Even for input data with larger detection error, the average accuracy rate of classification still achieves 75.52% in all the test experiments. It suggests that our model and algorithms are promising in classifying the distant multi-generation hybrid fishes.
2. Owing to the weakest intersection of highly expressed genes between BSB and TC, they are deterministically divided into two classes. However, there exists a higher transcript intersection between them and their hybrids. These findings have further deeply mined the biological genetic characteristics of distant hybridization generated by BSB and TC, based on optimization techniques and transcriptome data.
3. Although the hybrids of TC and BSB have been divided into different classes, the hybrids display higher transcript intersection. Since the transcript intersection of the hybrids and the parents is smaller than that among the hybrids, it can be concluded that the hybrid progeny of TC and BSB has significant hybrid characteristics, which may be useful to carry out trait improvement in practice.
4. Since $BT_{F_{3}}$ and $TB_{F_{3}}$ are classified to two different classes, where there only exist 3 shared genes of high expression, it is concluded that there exists larger trait separation in the third generation of TC and BSB hybrid progeny ($BT_{F_{3}}$ and $TB_{F_{3}}$). In other words, both $BT_{F_{3}}$ and $TB_{F_{3}}$ are a good variety for the reproduction of fish.
5. Since there are no shared genes of high expression between $BT_{F_{1}}$ and $TB_{F_{1}}$, they belong to two different classes (1st and 3rd classes). It implies that the reciprocal hybrids in the first generation of TC and BSB ($BT_{F_{1}}$ and $TB_{F_{1}}$) have larger biological distinction.

## Data Availability Statement

The genome assembly used in this study was downloaded from NCBI BioProject database (BioProject: http://www.ncbi.nlm.nih.gov/bioproject/) under accession numbers PRJNA269572. All raw mRNA-seq data were downloaded from the NCBI Sequence Read Archive (http://trace.ncbi.nlm.nih.gov/Traces/sra/) under accession number SRP050891.

## Author Contributions

ZW conceived and designed the study, wrote and revised the paper. JT designed and implemented the algorithm to analyze the data, and wrote the paper. LR, YX and SL did all the relevant experiments, collected the data and revised the paper.

## Funding

This research was supported by the National Science Foundation of China (Grant 71671190), National Key Research and Development Program of China (2018YFD0901202), National Science Foundation of China (31772902), and State Key Laboratory of Developmental Biology of Freshwater Fish (2018KF003).

## Conflict of Interest

The authors declare that the research was conducted in the absence of any commercial or financial relationships that could be construed as a potential conflict of interest.

## Appendices

### Results On Highly Expressed Genes

**Table A1:** A part of higly expressed genes of the six classes of fishes.

GeneID	NO	Elements in matrix *W* for each class					
		1st	2nd	3rd	4th	5th	6th
*Mam*27488	4285	8.04 x 10^-5^	0	0	0	0	0.2813
*Mam*12843	10739	5.91 x 10^-5^	0	0	0	0	0.279
*Mam*09635	15053	4.23 x 10^-4^	0	0	0	0	0.1783
*Mam*27746	109	0	0.4815	0	0	0	0.2766
*Mam*05349	1066	0	0.4789	0	0	0	0.1765
*Mam*04721	1278	0	0.4775	0	0	0	0.1254
*Mam*18643	1610	0	0.4789	0	0	0	0.1765
*Mam*26461	1720	0	0.4815	0	0	0	0.2766
*Mam*16947	2485	0	0.4815	0	0	0	0.2766
*Mam*03075	2654	0	0.4815	0	0	0	0.2766
*Mam*06110	2974	0	0.4815	0	0	0	0.2766
*Mam*21839	3102	0	0.4815	0	0	0	0.2766
*Mam*04828	3760	0	0.4815	0	0	0	0.2766
*Mam*08966	4306	0	0.4815	0	0	0	0.2766
*Mam*29639	4324	0	0.3065	0	0	0	0.2746
*Mam*11009	4467	0	0.4815	0	0	0	0.2766
*Mam*30659	5940	0	0.4815	0	0	0	0.2766
*Mam*10292	6294	0	0.4815	0	0	0	0.2766
*Mam*02487	7396	0	0.4815	0	0	0	0.2766
*Mam*07898	7412	0	0.4815	0	0	0	0.2766
*Mam*20311	7557	0	0.4815	0	0	0	0.2766
*Mam*05748	7783	0	0.4815	0	0	0	0.2766
*Mam*22143	8170	0	0.4815	0	0	0	0.2766
*Mam*16193	8446	0	0.4815	0	0	0	0.2766
*Mam*26424	8827	0	0.4815	0	0	0	0.2766
*Mam*25840	9858	0	0.4771	0	0	0	0.1103
*Mam*13519	10285	0	0.4815	0	0	0	0.2766
*Mam*25865	11901	0	0.4815	0	0	0	0.2766
*Mam*19044	12352	0	0.4815	0	0	0	0.2766
*Mam*16831	12585	0	0.4815	0	0	0	0.2766
*Mam*05543	13326	0	0.3065	0	0	0	0.2746
*Mam*13771	13506	0	0.4815	0	0	0	0.2766
*Mam*26854	13652	0	0.4815	0	0	0	0.2766
*Mam*00577	13715	0	0.4792	0	0	0	0.1905
*Mam*07942	14000	0	0.2847	0	0	0	0.2744
*Mam*07030	14089	0	0.4789	0	0	0	0.1765
*Mam*17634	14312	0	0.4815	0	0	0	0.2766
*Mam*18307	14829	0	0.4815	0	0	0	0.2766
*Mam*00814	15556	0	0.4789	0	0	0	0.1765
*Mam*10384	15720	0	0.4815	0	0	0	0.2766
*Mam*00295	16707	0	0.3065	0	0	0	0.2746
*Mam*11738	16870	0	0.4815	0	0	0	0.2766
*Mam*20672	17245	0	0.4815	0	0	0	0.2766
*Mam*27740	18056	0	0.4815	0	0	0	0.2766
*Mam*18895	18725	0	0.4815	0	0	0	0.2766
*Mam*22493	19575	0	0.3065	0	0	0	0.2746
*Mam*17452	19798	0	0.4815	0	0	0	0.2766
*Mam*00511	19852	0	0.4815	0	0	0	0.2766
*Mam*24132	6027	0	0	0	0.0342	0	0.2762
*Mam*14897	6151	0	0	0	0.0342	0	0.2762
*Mam*04754	6751	0	0	0	0.0342	0	0.2762
*Mam*00928	7106	0	0	0	0.0342	0	0.2762
*Mam*17991	8808	0	0	0	0.0342	0	0.2762
*Mam*05763	9053	0	0	0	0.0342	0	0.2762
*Mam*09936	10053	0	0	0	0.0342	0	0.2762
*Mam*09532	10428	0	0	0	0.0342	0	0.2762
*Mam*10304	12794	0	0	0	0.0342	0	0.2762
*Mam*19016	13794	0	0	0	0.0342	0	0.2762
*Mam*03189	16523	0	0	0	0.0342	0	0.2762

**Table A2:** The number of highly shared genes.

Relationship among $BSB^{L_{2}},\;TC^{L_{2}}$ and hybrids			
Relationship	Number	Relationship	Number
5*th* - 6*th* – 1*st*	107	5*th* - 6*th* – 2*st*	366
5*th* - 6*th* – 3*st*	58	5*th* - 6*th* – 4*st*	108
Relationship between $TC^{L_{2}}$ and hybrids		Relationship between $BSB^{L_{2}}$ and hybrids	
relationship	number	relationship	number
5*th* – 1*st*	0	6*th – *1*st*	3
5*th* – 2*nd*	0	6*th* – 2*nd*	45
5*th* – 3*rd*	0	6*th* – 3*rd*	0
5*th* – 4*^th^*	0	6*th* – 4*th*	12
5*th* – 1*st *– 2*nd *	5	6*st* – 1*st – *2*nd *	40
5*th* – 1*st *– 3*rd*	0	6*st* – 1*st* – 3*rd*	0
5*th* – 1*st *– 4*th*	0	6*st* – 1*st *– 4*th*	1
5*th* – 1*st *– 3*rd*	2	6*st* – 2*nd *– 3*rd*	13
5*th* – 2*nd *– 4*th*	587	6*st* – 2nd**– 4*th*	125
5*th* – 3*rd – 4th*	0	6*st* – 3*rd* – 4*th*	2
5*th* – 1*st *– 2*nd – *3*rd*	16	6*th* – 1*st – *2*nd – *3*rd*	27
5*th* – 1st – 2*nd *– 4*th*	483	6*th* – 1*st – *2*nd – *4*th*	168
5*th* – 1st – 3*rd *– 4*th*	1	6*th* – 1*st – *3*rd – *4*th*	6
5*th* – 2*nd* – 3*rd *– 4*th*	229	6*th* – 2*nd – *3*rd – *4*th*	88
5*th* – 1*^st^* **– 2*nd* – 3*rd *– 4*th*	2340	6*th* – 1*st – *2*nd – *3*rd – *4*th*	499
Relationship among hybrids			
Relationship	Number	Relationship	Number
1*st – *2*nd *	3	1*st* – 3*rd*	0
1*st – *4*th *	0	2*nd* – 3*rd*	7
2nd – 4*th*	0	3*rd – *4*th*	0
1*st – *2*nd – *3*rd*	4	1*st* – 2*nd* – 4*th*	277
1*st – *3*rd – *4*th*	0	2*nd* – 3*rd* – 4*th*	171
Other relationship			
1*st*	0	2*nd*	0
3*rd*	0	4*th*	0
5*th*	0	6*th*	0
5*th* – 6*th*	0	1*st – *2*nd – *3*rd – *4*th*	194

**Table A3:** A part of the base matrix *W *of the 1st-group samples *L_1_.*

GeneID	NO	Elements in base matrix *W* for each class					
		1st	2nd	3rd	4th	5th	6th
Mam01912	1	4.6868 x 10^-4^	0.0504	0.1706	0.0201	0.3837	9.3883 x 10^-6^
Mam21118	5	4.8315 x 10^-4^	5.7525 x 10^-4^	0.2237	7.2186 x 10^-4^	0	0
Mam11102	6	6.2526 x 10^-4^	0.0455	0.2249	0.0129	0.0745	0
Mam17081	7	3.7106 x 10^-4^	0.0671	0.1641	0.0389	0.0488	1.3303 x 10^-4^
Mam07456	8	5.2856 x 10^-4^	0.0398	0.0406	0.0509	0.3191	1.4597 x 10^-4^
Mam20030	9	4.2426 x 10^-5^	0.0411	0.1378	0.0614	0.3605	1.7073 x 10^-4^
Mam09854	10	1.3894 x 10^-4^	0.0300	0.2225	0.0552	0.1944	4.3024 x 10^-5^
Mam29205	13	1.5492 x 10^-4^	0.0072	0.1055	0.0185	0.4056	2.0497 x 10^-5^
Mam06683	14	3.0380 x 10^-4^	0.0530	0.0647	0.0470	0.3659	0
Mam19604	15	4.6509 x 10^-4^	0.0415	0.2180	0.0617	0.1979	0
Mam09824	16	1.2105 x 10^-5^	7.4416 x 10^-4^	0	6.2839 x 10^-4^	0.3519	0
Mam05355	18	2.1428 x 10^-4^	0.0662	0.2270	0.0496	0.3109	1.1562 x 10^-4^
Mam18093	19	3.2739 x 10^-5^	0.0264	0	0.0106	0.3837	6.1219 x 10^-5^
Mam23784	20	1.2477 x 10^-4^	0.0626	0.1883	0.0376	0.3666	1.1244 x 10^-4^
Mam16985	21	6.0934 x 10^-4^	6.7898 x 10^-4^	0.2239	8.5996 x 10^-4^	0	0
Mam02753	22	4.6572 x 10^-6^	0.0257	0.0570	0.0151	0.3913	1.2711 x 10^-4^
Mam23187	23	1.2105 x 10^-5^	7.4416 x 10^-4^	0	6.2839 x 10^-4^	0.3519	0
Mam05281	24	1.2105 x 10^-5^	7.4416 x 10^-4^	0	6.2839 x 10^-4^	0.3519	0
Mam28834	25	3.3784 x 10^-4^	0.0263	0.2251	0.0131	0.1040	0
Mam23819	26	3.1750 x 10^-4^	0.0668	0.1627	0.0500	0.1532	0
Mam07226	29	1.2105 x 10^-5^	7.4416 x 10^-4^	0	6.2839 x 10^-4^	0.3519	0
Mam11598	31	4.4154 x 10^-4^	0.0149	0.0745	0.0141	0.3357	0
Mam01497	32	2.5714 x 10^-4^	0.0327	0.1177	0.0195	0.4134	5.8852 x 10^-5^
Mam06448	33	5.0585 x 10^-6^	0.0313	0.0204	0.0512	0.1571	2.7127 x 10^-4^
Mam22869	35	2.2395 x 10^-4^	0.0187	0.1009	0.0178	0.3928	1.1210 x 10^-4^
Mam02037	36	2.1937 x 10^-4^	0.0180	0.0626	0.0269	0.3948	6.2672 x 10^-5^
Mam03780	37	0	0	0.0039	0	0.4115	2.4943 x 10^-5^
Mam23080	38	6.8878 x 10^-4^	0.0583	0.0636	0.0456	0.0646	9.1700 x 10^-5^
Mam23255	42	5.1783 x 10^-4^	8.9804 x 10^-4^	0.2263	0.0635	0.3869	2.3669 x 10^-4^
Mam18330	44	5.2189 x 10^-4^	0.0622	0.2187	0.0488	0	1.4495 x 10^-4^
Mam27424	45	0	0.0250	0.0786	0.0235	0.3616	6.7036 x 10^-5^
Mam22074	46	8.8226 x 10^-5^	0.0522	0.2247	0.0373	0.1112	0
Mam09837	47	5.6519 x 10^-4^	0.0404	0.1379	0	0	1.3783 x 10^-5^
Mam09179	49	1.5330 x 10^-4^	0.0433	0.0725	0	0.3250	2.1539 x 10^-4^
Mam11463	50	2.0732 x 10^-4^	0.0538	0.0675	5.8679 x 10^-4^	0.0547	2.5670 x 10^-4^
Mam28066	51	0	0	0.0110	0	0.4514	7.0319 x 10^-5^
Mam05693	52	0	0.0126	0.0025	0	0.4030	1.4919 x 10^-5^
Mam20805	53	1.2105 x 10^-5^	7.4416 x 10^-4^	0	6.2839 x 10^-4^	0.3519	0
Mam08145	54	3.1010 x 10^-4^	0.0512	0.0897	0.0244	0.3974	9.3436 x 10^-5^
Mam26031	55	1.6025 x 10^-4^	0.0060	0.0816	0.0211	0.4431	6.8717 x 10^-5^
Mam14647	56	5.9877 x 10^-4^	2.2448 x 10^-4^	0.1418	0.0139	0.3567	8.0921 x 10^-5^
Mam28671	57	2.9578 x 10^-4^	0.0422	0.1423	0.0630	0.2385	1.3530 x 10^-4^
Mam13535	58	5.4066 x 10^-5^	0.0172	0.0934	0.0274	0.3661	3.3498 x 10^-5^
Mam26404	61	2.0524 x 10^-4^	0.0110	0.0558	0	0.2177	1.4219 x 10^-4^
Mam28865	63	1.1933 x 10^-5^	0.0170	0.1906	0.0160	0.4052	1.0805 x 10^-4^
Mam14143	64	3.3864 x 10^-4^	0.0180	0.2079	0.0383	0.3853	0
Mam16854	65	0	0	0.0034	0	0.4082	2.1172 x 10^-5^
Mam22835	66	4.7533 x 10^-4^	1.3585 x 10^-4^	0.1844	0.0499	0.3343	1.7809 x 10^-4^
Mam05740	68	5.7847 x 10^-4^	0.0658	0.1137	0.0435	0.3608	0
Mam11399	69	4.5144 x 10^-4^	0.0660	0.2113	0.0175	0.0761	0
